# The presence of anhedonia in individuals with subacute and chronic stroke: an exploratory cohort study

**DOI:** 10.3389/fnagi.2024.1253028

**Published:** 2024-02-07

**Authors:** Emma Segura, Adrià Vilà-Balló, Aida Mallorquí, María F. Porto, Esther Duarte, Jennifer Grau-Sánchez, Antoni Rodríguez-Fornells

**Affiliations:** ^1^Cognition and Brain Plasticity Unit, Bellvitge Biomedical Research Institute, L’Hospitalet de Llobregat, Barcelona, Spain; ^2^Department of Cognition, Development and Educational Psychology, University of Barcelona, Barcelona, Spain; ^3^Institute of Neurosciences, University of Barcelona, Barcelona, Spain; ^4^Clinical Health Psychology Section, Clinic Institute of Neuroscience, Hospital Clínic, Barcelona, Spain; ^5^Department of Physical and Rehabilitation Medicine, Hospital del Mar, Barcelona, Spain; ^6^Institut Hospital del Mar d'Investigacions Mèdiques (IMIM), Barcelona, Spain; ^7^Research Group on Complex Health Diagnoses and Interventions from Occupation and Care (OCCARE), Escola Universitària d'Infermeria i Teràpia Ocupacional, Autonomous University of Barcelona, Barcelona, Spain; ^8^Institució Catalana de Recerca i Estudis Avançats, Barcelona, Spain

**Keywords:** subacute and chronic stroke, anhedonia, depression, motivation, rehabilitation

## Abstract

**Background:**

Anhedonia refers to the diminished capacity to experience pleasure. It has been described both as a symptom of depression and an enduring behavioral trait that contributes its development. Specifically, in stroke patients, anhedonia has been closely linked to depression, resulting in reduced sensitivity to everyday pleasures and intrinsic motivation to engage in rehabilitation programs and maintain a healthy active lifestyle. This condition may hinder patients’ recovery, diminishing their autonomy, functioning, and quality of life.

**Objective:**

We aimed to explore the prevalence and level of anhedonia and those variables that might be associated in patients with both ischemic and hemorrhagic stroke at subacute and chronic phases of the disease.

**Methods:**

We conducted an exploratory cohort study with a sample of 125 patients with subacute and chronic stroke presenting upper-limb motor deficits. We measured participants’ level of anhedonia with four items from the Beck Depression Inventory-II that describe the symptoms of this condition: loss of pleasure, loss of interest, loss of energy, and loss of interest in sex. We also collected demographic and clinical information and evaluated motor and cognitive functions as well as levels of depression, apathy, and various mood states. The results were compared to a sample of 71 healthy participants of similar age, sex, and level of education.

**Results:**

Stroke patients demonstrated a significantly higher prevalence (18.5–19.7%) and level of anhedonia compared to the healthy controls (4.3%), regardless of stroke phase, level of motor impairment, and other clinical variables. Furthermore, post-stroke anhedonia was associated with lower levels of motivation and higher levels of negative mood states such as fatigue and anger in the long term. Importantly, anhedonia level was superior in stroke patients than in healthy controls while controlling for confounding effects of related emotional conditions.

**Conclusion:**

This study provides novel evidence on the prevalence, level and factors related to anhedonia post-stroke. We emphasize the importance of assessing and treating anhedonia in this population, as well as conducting large-scale cohort and longitudinal studies to test its influence on long-term functional and emotional recovery.

## 1 Introduction

Anhedonia is defined as the diminished capacity to experience pleasure. The concept was rarely used before the 1980s and was officialized in the DSM-III, becoming a necessary symptom for depression diagnosis (Der-Avakian and Markou, 2012). In 2008, the American Psychiatric Association developed the Research Domain Criteria framework to integrate transdiagnostic neurobehavioral evidence in the study of mental disorders, where anhedonia was considered a behavioral correlate of the negative valence human systems domain (Cuthbert, 2021). Anhedonia has also been addressed as a residual symptom of schizophrenia (Der-Avakian and Markou, 2012), but most recently, it was included in the DSM-V as a crucial symptom for diagnosing the major depression melancholic subtype and differentiating it from other mental disorders (American Psychiatric Association, 2013).

Besides the medical symptom approach, anhedonia has been described as an enduring and stable behavioral trait that characterizes an individual’s personality (Der-Avakian and Markou, 2012). The manifestation of anhedonia has been associated with deficits in the reward processing system, specifically in the dopaminergic projections between the midbrain dopaminergic nucleus, striatum, amygdala, hippocampus, and prefrontal cortex, requested for modulating behavioral responses to rewards (Camara et al., 2009; Der-Avakian and Markou, 2012). Previous studies have suggested the genetic influence of developing anhedonia by demonstrating genetic polymorphisms of dopamine synthesis, metabolism, and regulator proteins that impair functional activity of brain regions belonging to the reward system (Ren et al., 2018). Accordingly, it has been proposed the potential endophenotype role of anhedonia as a vulnerability marker present before the onset of depression that might increase the severity and prolong the course of this mood disorder (Gong et al., 2017). Furthermore, suffering depression with severe symptoms of anhedonia has also been associated with an elevated risk of suicide (Bonanni et al., 2019). Individuals with high anhedonia-trait levels could thus be more likely to develop depression in a particular context (Pizzagalli et al., 2005; Auerbach et al., 2019). For example, anhedonia has been associated with increased levels of depression at hospital discharge in stroke patients (Sibon et al., 2012). In this regard, the disruption of dopaminergic networks after a stroke could trigger the onset of mood disorders and cognitive deficits including memory dysfunction, leading to the development of anhedonia (Piamarta et al., 2004; Terroni et al., 2015).

In addition to mental health disorders, anhedonia has been found in other health conditions involving chronic pain and inflammatory processes (Carpinelli et al., 2019; Lucido et al., 2021). For instance, a control–case study reported higher anhedonia levels in chronic pain sufferers when compared to healthy controls, with 25% of patients obtaining scores over the standard cutoff point (Garland et al., 2020). Interestingly, this result was not entirely motivated by a comorbid depression diagnosis, pointing out a more nuanced perspective of the psychological consequences of some health conditions. Furthermore, a recent review on endometriosis, a gynecological chronic systemic inflammatory disease characterized by the presence of chronic pelvic pain, presented anhedonia as a severe symptom which is not assessed in the usual clinical treatment despite the deleterious consequences in patients’ mental health and quality of life (QoL) (Mallorqui et al., 2022).

Stroke is the most prevalent neurological disease and a leading cause of acquired long-term disability worldwide (Katan and Luft, 2018; Feigin et al., 2019). Motor, cognitive, and language deficits are common post-stroke consequences that limit the accomplishment of daily activities and restrict participation in familiar, social, work, and community life (Hartman-Maeir et al., 2007; Tang et al., 2020). The reduction of the functional autonomy of stroke survivors has negative effects on their emotional well-being and QoL, leading to mood disorders such as depression and anxiety (Patel et al., 2002; Marcheschi et al., 2018; Diamond et al., 2023). Importantly, most patients still present or even increase motor deficits in the chronic phase of the disease (Kwakkel et al., 2003). Once patients complete formal rehabilitation programs, a decrease in physical activity mostly attributed to a lack of a rehabilitation routine and/or therapist presence can provoke a decline in motor functions (Ashe et al., 2009). This underscores the importance of intrinsic motivation in sustaining a healthy and active lifestyle (Chan et al., 2009).

Stroke survivors pass through challenging periods in which they need to adapt their lives to their new condition. Between 25 and 79% of stroke patients suffer post-stroke depression (PSD), whose cause is associated with the physical and psychological adversities they must cope with (Whyte and Mulsant, 2002). Previous research have demonstrated the negative impact of PSD on motor and cognitive recovery, becoming an additional disabling factor responsible for 15% of increased disability (Paolucci et al., 2019). This detrimental consequence has been associated with deficits in regulating motivation-related behaviors (Gainotti et al., 2001). Many studies have focused on exploring the negative impact of apathy on stroke rehabilitation (Marin, 1991; Sibon et al., 2012), but motivational mechanisms and goal-directed behaviors are also disrupted by anhedonia, an understudied condition in the stroke population. Despite the substantial overlap between apathy and anhedonia, the former is associated with an impairment in selecting future behaviors based on emotional signals, while anhedonia is characterized by diminishing sensitivity to everyday pleasures and positive mood, crucial aspects for goal-directed engagement in rewarding activities (Verrienti et al., 2023). Considering the role of anhedonia in motivational aspects crucial for stroke survivors’ recovery, a recent study by Ashaie et al. (2023) explored the association between three dimensions of PSD somatic symptoms, negative affect, and anhedonia. They found that anhedonia predicted subsequent increases in somatic symptoms during the first year after rehabilitation discharge, indicating its impact on physical distress rather than stroke itself. In this regard, the authors emphasized the importance of examining specific dimensions of PSD, such as anhedonia, to understand their development and etiology and thus be able to guide clinicians in implementing targeted treatments and improving therapeutic outcomes (Ashaie et al., 2023). Currently, no previous study has examined the presence of anhedonia in patients with both ischemic and hemorrhagic stroke and over a wide range of time post-stroke.

In the present exploratory cohort study, we aimed to determine the prevalence and levels of anhedonia in subacute and chronic stroke survivors with upper-limb motor deficits, and to explore the factors associated with this condition in the stroke population. We hypothesized that two groups of subacute and chronic stroke patients would show a higher prevalence and level of anhedonia than a group of healthy individuals, and that anhedonia would be related to negative mood states. A better understanding of the onset of anhedonia post-stroke and its related factors would help design more effective and evidence-based interventions aimed to improve patients’ emotional well-being and promote adherence to rehabilitation and healthy active lifestyles.

## 2 Materials and methods

### 2.1 Participants

Two groups of patients, one with subacute stroke (SS) (*n* = 54; females = 24; age = 61.44 ± 8.72 years; time since stroke = 2 ± 1.5 months) and another with chronic stroke (CS) (*n* = 71; females = 17; age = 62.27 ± 11.40; time since stroke = 20.75 ± 48.63 months), were included in the present study. All patients were recruited from the Physical Medicine and Rehabilitation Department of Hospital del Mar, Centre l’Esperança (Barcelona, Spain) to participate in experimental studies or randomized controlled trials that aimed to test the effectiveness of music-based interventions in the improvement of upper-limb motor functions. As part of these studies, patients underwent a basal evaluation that included motor, cognitive, emotional well-being and QoL assessment (Ripollés et al., 2016; Grau-Sánchez et al., 2018, 2021).

The studies were conducted by a group of research assistants that included psychologists and occupational therapists with training in clinical research. The inclusion criteria for both samples of stroke patients were: (1) mild-to-moderate paresis of the upper extremity after a first-ever stroke; (2) no major cognitive deficits affecting comprehension; (3) no neurological or psychiatric co-morbidity, except for PSD; (4) no other musculoskeletal condition affecting upper extremity motor function (e.g., fracture or arthritis); (5) ability to speak Spanish and/or Catalan. The specific inclusion criteria for the subacute sample were (1) less than 6 months after the stroke and (2) being involved in a program of outpatient rehabilitation at the Department of Physical Medicine and Rehabilitation at the Hospital del Mar, Centre l’Esperança. The specific inclusion criteria for the chronic stroke sample (1) more than 6 months post-stroke, and (2) have previously completed a 6-month formal rehabilitation program.

A group of healthy participants (HC) (*n* = 70; females = 31; age = 59.31 ± 13.81 years) who never suffered a stroke was recruited as a control group. The inclusion criteria were: (1) no presence of paresis or any musculoskeletal condition affecting upper-limb motor function; (2) no major cognitive deficits affecting comprehension; (3) no neurological or psychiatric co-morbidity; (4) ability to speak Spanish and/or Catalan. When recruiting the control sample, we considered their age, sex, and level of education to obtain similar groups in terms of demographic characteristics. They were recruited through the dissemination of the study in social networks, among relatives of research assistants, and from a residential service of temporary stays for elderly people called *Respir* in Barcelona.

### 2.2 Assessment

Demographic and clinical variables such as age, sex, stroke etiology, affected hemisphere, lesion location, and time since stroke were collected from medical records. A structured interview was conducted at the hospital to assess patients’ upper-limb paresis and global cognitive function and check if they fulfilled the inclusion criteria. Paresis level was measured using the Medical Research Council Scale at the distal muscles of the upper extremity. Global cognitive function was measured using the Spanish version of the Mini-Mental State Examination (MMSE, Folstein and Folstein, 1975; Lobo, 1999) in subacute stroke patients, chronic stroke patients recruited by Ripollés et al. (2016), and healthy control participants; and the Spanish version of Montreal Cognitive Assessment (MoCA, Nasreddine et al., 2005; Gallego et al., 2009) in chronic stroke patients recruited by Grau-Sánchez et al. (2021). The presence of mild cognitive impairment (MCI) was defined following the cutoffs used in Pendlebury et al. (2012), indicating MCI when scoring lower than 27 in MMSE; and the cutoffs established by Pereiro et al. (2017), indicating the presence of MCI when scoring lower than 26 in MoCA.

Those patients who fulfilled the inclusion criteria were invited to attend another day at the hospital to complete the baseline evaluation. It consisted of a one-hour session of motor and cognitive functions assessments and was conducted by research members blinded to the intervention group. Furthermore, self-report emotional and QoL questionnaires were given to patients to complete at home (Ripollés et al., 2016; Grau-Sánchez et al., 2018, 2021). The motor, cognitive and emotional evaluation of healthy controls participants was performed entirely in person at their home or remotely via the Zoom platform depending on their preference.

#### 2.2.1 Motor evaluation

Upper-limb functional movements were evaluated using the Action Research Arm Test (ARAT) (Lyle, 1981). The ARAT has excellent test–retest and inter/intra-rater reliability (Van Der Lee et al., 2001; Platz et al., 2005) and it is recommended for use in chronic stroke and outpatient rehabilitation by the StrokeEDGE Task Force Group (Sullivan et al., 2013). The test consists of 19 items divided into four subtests: grasp, grip, pinch, and gross movement. For each item, the patient is asked to perform a simple task involving a functional movement of the affected upper-limb. Each task is rated with a 4-point ordinal scale (from 0: “*not possible to perform the task*” to 3: “*performing the task normally*”). The minimum score is 0 and the maximum is 57, with higher scores indicating a higher level of upper-limb functionality.

#### 2.2.2 Cognitive evaluation

Working memory and attention were assessed using the Digit Span (forward and backward) subtest from the Wechsler Adult Intelligence Scale III (WAIS-IV, Wechsler, 2013). It consists of two parts: Digit Span Forward, in which participants are asked to repeat aloud a series of digits in the same order that gradually increases until the individual is unable to repeat the sequence; and Digit Span Backward, with the same procedure but the digits must be repeated in the reverse order. Scores are based on the longest length of sequence repeated correctly for each part. The minimum score is 0 and the maximum scores are 16 and 14 in the Forward and Backward parts, respectively. Raw scores were transformed into normative data considering individuals’ age according to Wechsler (2013), with higher scores indicating a higher capacity of attention and working memory.

Verbal learning and memory abilities were measured using the Spanish Version of the Rey Auditory Verbal Learning Test (RAVLT) (Marqués et al., 2013). In this test, participants are asked to listen and memorize a list of 15 unrelated words and immediately recall them for a total of five trials. Then, an interference list of 15 different unrelated words is presented and participants are asked to recall as many words as possible. After a 20-min delay, participants are asked to recall as many words as possible from the first list, and to complete a recognition task on both lists with distractors. For each task, the minimum score is 0 and the maximum is 15. The total sum words of the first five trials (0–75 score) were transformed into normative data considering individuals’ age according to Stricker et al. (2021), with higher scores indicating a higher capacity of verbal memory.

#### 2.2.3 Emotional and mood evaluation

Depression was assessed using the Spanish version of the Beck Depression Inventory-II (BDI-II) (Beck et al., 1996; Sanz et al., 2003), a self-report measure comprised of 21 multiple-choice questions scored from 0 to 3 about patients’ feelings, thoughts, and behaviors over the past week. The minimum score is 0 and the maximum is 63, with higher scores indicating depression severity. The level of depression was defined following the cutoffs established by Beck and Beamesderfer (1974) for the stroke population: no depression (0–9 scores), mild depression (10–18 scores), moderate depression (19–29 scores), and severe depression (30–63 scores).

Anhedonia was assessed by calculating the total sum score of four items from the BDI-II that describe the symptoms of this behavioral trait according to Pizzagalli et al. (2005): loss of pleasure (item 4), loss of interest (item 12), loss of energy (item 15), and loss of interest in sex (item 21). The minimum score is 0 and the maximum is 12, with higher scores indicating higher anhedonia. Participants were classified into higher or lower levels of anhedonia following the cutoff obtained from calculating the 95% distribution value of the anhedonia BDI-II subscale score in HC, which was 4. Participants who obtained an anhedonia score greater than 4 were classified as higher anhedonic (HAnh), while those who obtained a score equal to or lower than 4 were classified as lower anhedonic (LAnh).

A non-anhedonic component of depression was calculated by subtracting the anhedonia BDI-II subscale score from the BDI-II total score, in order to explore the relationship between anhedonia and the non-anhedonic component of depression. The minimum score is 0 and the maximum 51, with higher scores indicating higher non-anhedonic depression. Individuals were classified into higher and lower levels of non-anhedonic depression following the cutoff obtained from calculating the 95% distribution value of the non-anhedonic depression BDI-II subscale score in HC, which was 16.65. Participants who obtained a non-anhedonic depression score greater than 16.65 were classified as higher non-anhedonic depressed (HDep), while those who obtained a score equal to or lower than 16.65 were classified as lower non-anhedonic depressed (LDep).

Apathy was evaluated using the Self-Rated Version of the Apathy Evaluation Scale (AES-S) and the Informant Version of the Apathy Evaluation Scale (AES-I), both translated into Spanish (Marin, 1991). The AES-S and AES-I consist of 18 items scored on a 4-point Likert scale (from 1: “*a lot*” to 4: “*nothing*”) about behavioral, cognitive, and emotional aspects of patients’ apathy over the past 4 weeks. The minimum score is 18 and the maximum 72, with higher scores indicating more apathy. The presence of apathy was defined following the cutoffs established by Andersson et al. (1999), indicating the presence of apathy in the stroke population when scoring equal or higher than 34 in both self- and informant-versions of the apathy scale (AES-S and AES-I).

Different dimensions of mood such as anger, vigor, fatigue, confusion, tension and depression levels were assessed with the Profile of Mood States (POMS) by asking the participant to rate feelings or emotions felt over the past week (McNair et al., 1971). This measure includes 65 items scored on a 5-point Likert scale (from 0: “*not at all*” to 4: “*extremely*”) and classified into the six subscales. The minimum score is 0 in all subscales, and the maximum scores are 48 in Anger-Hostility, 32 in Vigor-Activity and Tension-Anxiety, 28 in Fatigue-Inertia, 24 in Confusion-Bewilderment, and 60 in Depression-Dejection. Higher scores indicate higher levels of each mood dimension.

### 2.3 Statistical analysis

#### 2.3.1 Descriptive analysis

Absolute frequencies and percentages were calculated for the categorical variables: sex, stroke etiology, affected hemisphere, lesion location, global cognitive function level (MCI < 27 in MMSE; MCI < 26 in MoCA), depression level (none: 0–9 scores; mild: 10–18 scores; moderate: 19–29 scores; severe: 30–63 scores), anhedonia level (HAnh >4; LAnh ≤4), non-anhedonic depression level (HDep >16.65; LDep ≤16.65), and presence of apathy (scoring ≥34 in AES-S and AES-I). The mean and standard deviation (SD) were calculated for the quantitative variables with a parametric distribution: age, Digit Span, RAVLT, and Vigor-POMS score. The median and the interquartile range (IQR) were calculated for the quantitative variables with a nonparametric distribution: years of education, months post-stroke, and the scores on ARAT, MMSE, MoCA, anhedonia BDI-II subscale, AES-S, AES-I, BDI-II, non-anhedonic depression BDI-II subscale, and all POMS subscales. Regarding the level of global cognitive function, we combined MMSE and MoCA scores due to their strong correlation (*r* = 0.79) in subacute stroke patients (Toglia et al., 2011).

The differences between the three groups (SS, CS, and HC) were evaluated with two-way ANOVA test and Kruskal–Wallis test for independent samples for quantitative variables with parametric and nonparametric distribution, respectively, and Fisher’s exact test for categorical variables. Post-hoc analysis was conducted using Tukey’s and Dunn’s tests for continuous variables with parametric and nonparametric distribution, respectively, applying Bonferroni correction for multiple comparisons. We used Fisher’s exact test for pairwise comparisons of categorical variables between groups.

Considering that anhedonia level did not differ between subacute and chronic samples (see Descriptive analysis in Results), a secondary analysis was performed to explore the contribution of anhedonia to different factors in the stroke population. Thus, demographic, clinical, and emotional outcomes were compared between stroke patients classified as HAnh (scoring >4 in anhedonia BDI-II subscale) and LAnh (scoring ≤4 in anhedonia BDI-II subscale).

#### 2.3.2 Correlation analysis

The anhedonia score was correlated with continuous demographic, clinical, and emotional variables to further explore which factors were related to anhedonia levels in both groups of patients (SS and CS). We used Spearman’s test to check correlations since anhedonia scores showed a nonparametric distribution.

Due to the strong correlation between anhedonia and non-anhedonic depression, partial correlation analyses were applied to explore the relationship between the anhedonia and the continuous demographic, clinical and emotional variables while controlling the influence of the non-anhedonic depression BDI-II subscale in subacute and chronic stroke patients, as well as in the whole sample of patients. Based on the strong correlation between anhedonia and the other emotional variables in both SS and CS groups (see Correlation analysis in Results), an additional analysis was performed comparing anhedonia level between stroke patients and the HC group while controlling for the confounding effects of total BDI-II score, non-anhedonic BDI-II subscale score, AES-S and AES-I scores, and all POMS subscales scores.

The descriptive and correlation statistical analyses were conducted using the R (version 4.2.2) and RStudio (version 2022.12.0 + 353), and the level of significance was set at 0.05.

## 3 Results

### 3.1 Descriptive analysis

There were no significant differences in age, years of education, stroke etiology, and affected hemisphere between groups (see Table 1). The SS showed a major distribution of stroke lesions in cortico-subcortical regions compared to the CS, which showed a major distribution in subcortical areas. Post-hoc analysis (see

**Table 1:** Descriptive analysis of demographic and clinical variables.

	Subacute group *N* = 54	Chronic group *N* = 71	Healthy group *N* = 70	Value of *p*
**Demographic variables**				
Age	61.44 (8.72)	62.27 (11.40)	59.31 (13.81)	0.379
*Sex*				
Females	24 (44.44%)	17 (23.94%)	31 (44.29%)	0.016*
Males	30 (55.56%)	54 (76.06%)	39 (55.71%)	
*Education level*				
Years of education	18 [5.25]	18 [10]	18 [13]	0.266
**Clinical variables**				
*Stroke etiology*				
Ischemic	42 (77.78%)	46 (64.79%)		0.117
Hemorrhagic	12 (22.22%)	25 (35.21%)		
*Affected hemisphere*				
Right	24 (44.44%)	37 (52.11%)		0.471
Left	30 (55.56%)	34 (47.89%)		
*Lesion location*				
Cortical	2 (3.70%)	10 (14.08%)		<0.001*
Cortico-subcortical	34 (62.96%)	16 (22.54%)		
Subcortical	13 (24.07%)	34 (47.89%)		
Brainstem	2 (3.70%)	7 (9.86%)		
Cerebellum	3 (5.56%)	4 (5.63%)		
*Time since stroke*				
Months post-stroke	2 [1.50]	20.75 [48.63]		<0.001*
*Motor ability*				
ARAT	41.50 [21.75]	42 [15]	57 [0]	<0.001*
*Cognitive level*				
MoCA/MMSE score	27 [3]	27 [4.50]	30 [1.75]	<0.001*
No impairment (%)	32 (59.25%)	45 (63.38%)	62 (88.57%)	<0.001*
Mild impairment (%)	22 (40.74%)	26 (36.62%)	8 (11.43%)	
*Memory*				
Digit span (normative) score	10.02 (2.86)	11.77 (2.90)	12.27 (2.60)	<0.001*
RAVLT (normative) score	114.11 (30.13)	126.65 (40.5)	127.99 (44.10)	0.076

Absolute frequencies and percentages are shown for the variables sex, stroke etiology, affected hemisphere, lesion location, and cognitive level. The mean and the standard deviation (SD) or the median and interquartile ranges [IQR] are shown for the quantitative variables with parametric and nonparametric distribution, respectively. *p* < 0.05. Mild impairment < 27 in MMSE and < 26 in MoCA. ARAT, Action Research Arm Test; MoCA, Montreal Cognitive Assessment; MMSE, Mini-Mental State Examination; RAVLT, Rey Auditory Verbal Learning Test.

Table 2) revealed significant differences in sex between the CS and the other two groups, but all showed a greater percentage of men. The HC showed higher levels of motor and global cognitive functions when compared to SS and CS, who showed no differences between them. The HC showed 11.43% of individuals with MCI (MCI < 27 in MMSE; MCI < 26 in MoCA), compared to 40.74 and 36.62% in SS and CS, respectively. Finally, SS demonstrated lower scores in Digit Span than CS and HC.

**Table 2:** Post-hoc analysis of demographic and clinical variables.

Demographic and clinical variables	*p*-value of group comparisons				
	Sex	ARAT score	MMSE/MoCA score	Cognitive impairment	Digit span score
Subacute group	Chronic group	<0.021^*^	>0.999	<0.550	<0.710	<0.002^*^
Healthy group	Subacute group	>0.999	<0.001^*^	<0.001^*^	<0.001^*^	<0.001^*^
	Chronic group	<0.021^*^	<0.001^*^	<0.001^*^	<0.001^*^	<0.540

*p*-value of pairwise comparisons are shown for those variables that differed significantly between groups in Table 1. *p* < 0.05. ARAT, Action Research Arm Test; MMSE, Mini-Mental State Examination; MoCA, Montreal Cognitive Assessment.

There were significant differences between groups in all emotional variables, except for the classification of non-anhedonic depression level (HDep >16.65 in non-anhedonic depression BDI-II subscale) and for the POMS, where groups only differed in Vigor-Activity subscale (see Table 3). Post-hoc analysis revealed that all significant differences were between HC and both groups of patients, who did not differ between them (see

**Table 3:** Descriptive analysis of emotional variables.

	Subacute group *N* = 54	Chronic group *N* = 71	Healthy group *N* = 70	*p-*value
**Emotional variables**				
*Anhedonia*				
Anhedonia score	2 [3]	3 [3]	1 [2]	<0.001^*^
Lower anhedonia	44 (81.48%)	57 (80.28%)	67 (95.71%)	0.009^*^
Higher anhedonia	10 (18.52%)	14 (19.72%)	3 (4.29%)	
*Depression*				
BDI-II score	9 [9.75]	11 [10.50]	4 [4.75]	<0.001^*^
None	28 (51.85%)	30 (42.25%)	59 (84.29%)	<0.001^*^
Mild	16 (29.63%)	27 (38.03%)	7 (10%)	
Moderate	8 (14.81%)	8 (11.27%)	3 (4.29%)	
Severe	2 (3.7%)	6 (8.45%)	1 (1.43%)	
Non-anhedonic BDI-II score	7 [7.5]	10 [8]	3 [3]	<0.001^*^
Lower non-anhedonic depression	45 (83.33%)	59 (83.10%)	66 (94.29%)	0.074
Higher non-anhedonic depression	9 (16.67%)	12 (16.90%)	4 (5.71%)	
*Apathy informed by patient*				
AES-S score	34 [8]	36 [10.5]	31 [8]	<0.001^*^
Presence	28 (52.83%)	44 (61.97%)	21 (30%)	<0.001^*^
No presence	25 (47.17%)	27 (38.03%)	49 (70%)	
*Apathy informed by caregiver*				
AES-I score	34 [11.75]	35 [14.50]	28 [8]	<0.001^*^
Presence	28 (52.83%)	40 (56.34%)	15 (23.08%)	<0.001^*^
No presence	25 (47.17%)	31 (43.66%)	50 (76.92%)	
*POMS*				
Anger-Hostility score	6 [8]	6 [11]	6 [7.75]	0.853
Vigor-Activity score	14.64 (5.14)	13.41 (5.39)	17.04 (4.97)	<0.001^*^
Fatigue-Inertia score	6 [10]	5 [8]	6 [8]	0.691
Tension-Anxiety score	7 [10]	6 [8.50]	7.50 [9]	0.756
Confusion-Bewilderment score	3 [7]	3 [10]	2 [7]	0.916
Depression-Dejection score	8 [12]	9 [16.50]	6 [8.75]	0.174

All participants completed the whole evaluation, except for one SS participant who did not respond the AES-S and POMS; and one SS and five HC participants who did not provide the AES-I. Spouses mainly answered the AES-I, followed by siblings, children, and caregiver. Absolute frequencies and percentages are shown for the level of anhedonia, presence of apathy, and level of depression. The mean and the standard deviation (SD) or the median and interquartile ranges (IQR) are shown for the quantitative variables with parametric and nonparametric distribution, respectively. *p* < 0.05. BDI-II, Beck Depression Inventory-II; AES-S, Self-Rated Version of the Apathy Evaluation Scale; AES-I, Informant Version of the Apathy Evaluation Scale; POMS, Profile of Mood States; none depression: 0–9 scores in BDI-II; mild depression: 10–18 scores in BDI-II; moderate depression: 19–29 scores in BDI-II; severe depression: 30–63 scores in BDI-II; lower anhedonia ≤ 4 in anhedonia BDI-II subscale; Higher anhedonia > 4 in anhedonia BDI-II subscale; lower non-anhedonic depression ≤ 16.65 in non-anhedonic depression BDI-II subscale; higher non-anhedonic depression > 16.65 in non-anhedonic depression BDI-II subscale; presence of apathy ≥ 34 in AES-S and AES-I.

Table 4). The SS and CS groups showed a significantly higher anhedonia score than HC (see

**Table 4:** *Post-hoc* analysis of emotional variables.

Emotional variables		*p-*values of group comparisons					Anhedonia score	Higher anhedonia presence	BDI-II score	Non-anhedonic BDI-II score	Depression level
Subacute group	Chronic group	<0.293	>0.999	<0.774	>0.989	<0.474
Healthy group	Subacute group	<0.001^*^	<0.016^*^	<0.001^*^	<0.001^*^	<0.001^*^
	Chronic group	<0.001^*^	<0.008^*^	<0.001^*^	<0.001^*^	<0.001^*^

		AES-S score	Apathy presence by patient	AES-I score	Apathy presence by caregiver	Vigor (POMS) score
Subacute group	Chronic group	<0.129	<0.359	<0.456	<0.854	<0.389
Healthy group	Subacute group	<0.029^*^	<0.015^*^	<0.001^*^	<0.002^*^	<0.031^*^
	Chronic group	<0.001^*^	<0.001^*^	<0.001^*^	<0.001^*^	<0.001^*^

*p-*values of pairwise comparisons are shown for those variables that differed significantly between groups in Table 3. *p* < 0.05. BDI-II, Beck Depression Inventory-II; AES-S, Self-Rated Version of the Apathy Evaluation Scale; AES-I, Informant Version of the Apathy Evaluation Scale; POMS, Profile of Mood States.

**Figure 1:**
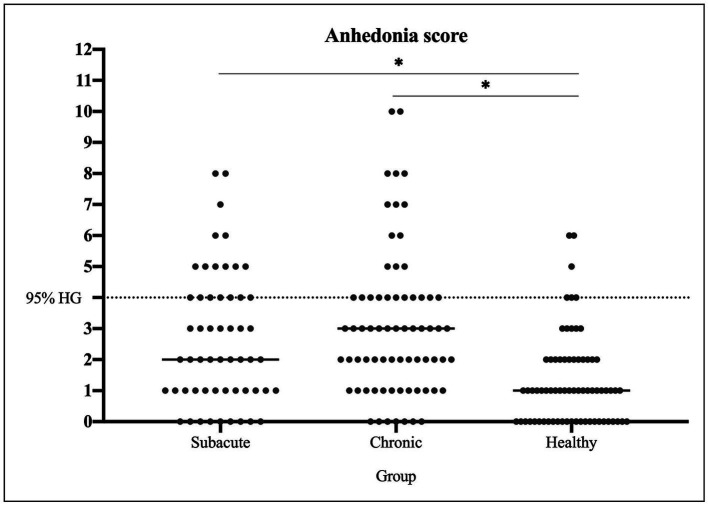
Anhedonia distribution between groups. Anhedonia score is shown for all individuals of each group: subacute stroke, chronic stroke, and healthy controls.

**Figure 2:**
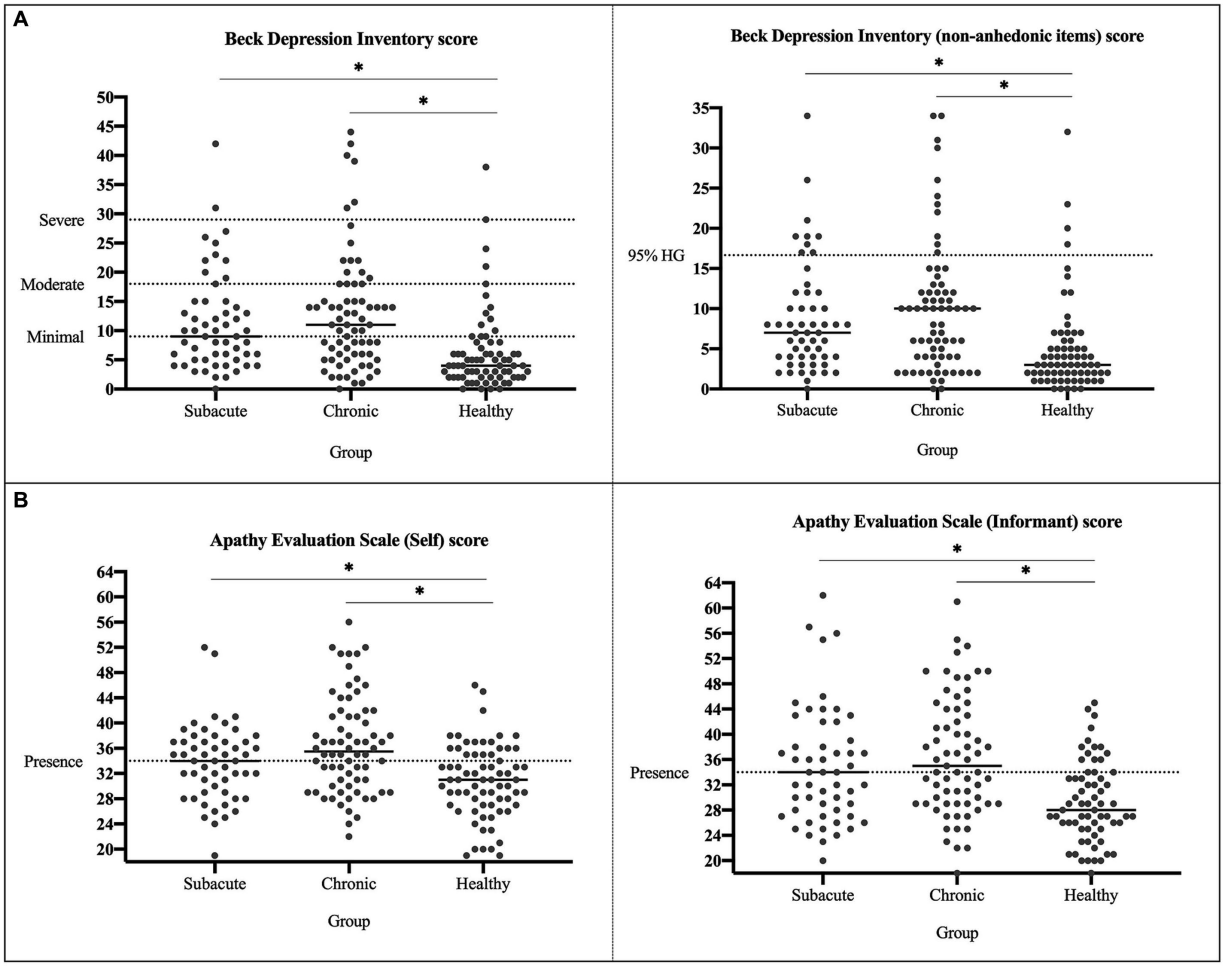
Apathy and depression distribution between groups. **(A)** Apathy scores of both self- and informant-versions are shown for all individuals from each group. **(B)** Depression scores with all the items and without the anhedonia items are shown for all individuals from each group.

Figure 1). Compared to 4.29% of HC exhibiting HAnh (scoring >4 in anhedonia BDI-II subscale), 18.52% of SS and 19.72% of CS patients showed a clear presence of anhedonia. The SS and CS also obtained a significantly higher score in apathy scales (AES-S and AES-I), BDI-II, and non-anhedonic depression BDI-II subscale. A greater percentage of patients reached the cutoff point for apathy presence (scoring ≥34 in AES-S and AES-I) (SS: 52.83% in self- and informant-versions; CS: 61.97% in self-version and 56.34% in informant-version) compared to HC (30% in self-version and 23.08% in informant-version) (see Figure 2A). A greater percentage of patients also showed moderate (19–29 scores in BDI-II) or severe (30–63 scores in BDI-II) level of depression (SS: 18.51%; CS: 19.72%) compared to HC (5.72%) (see Figure 2B). Finally, the HC scored significantly higher on the Vigor-Activity POMS compared to both SS and CS.

When comparing demographic variables between stroke patients with different anhedonia level, HAnh patients (scoring >4 in anhedonia BDI-II subscale) showed significantly lower years of education than LAnh patients, while no differences were found on any clinical variable. At the emotional level, HAnh patients reported significantly higher levels of depression, apathy (AES-S and AES-I), Anger-Hostility, Fatigue-Inertia, Tension-Anxiety, and Confusion-Bewilderment, and significantly lower levels of Vigor-Activity compared to LAnh patients. While 70.83% of HAnh patients showed moderate and severe levels of depression (19–63 scores in BDI-II), with, 93.07% of LAnh patients demonstrated no or mild depression (0–18 scores in BDI-II), with none of them reaching the severe level. A significantly larger percentage of relatives reported presence of apathy (scoring ≥34 in AES-I) in HAnh patients (79.17%) compared to LAnh patients (49.50%). Interestingly, no differences were found in the proportion of patients classified as HDep (scoring >16.65 in non-anhedonic depression BDI-II subscale) and LDep between groups (see Supplementary Table S1).

### 3.2 Correlation analysis

**Figure 3:**
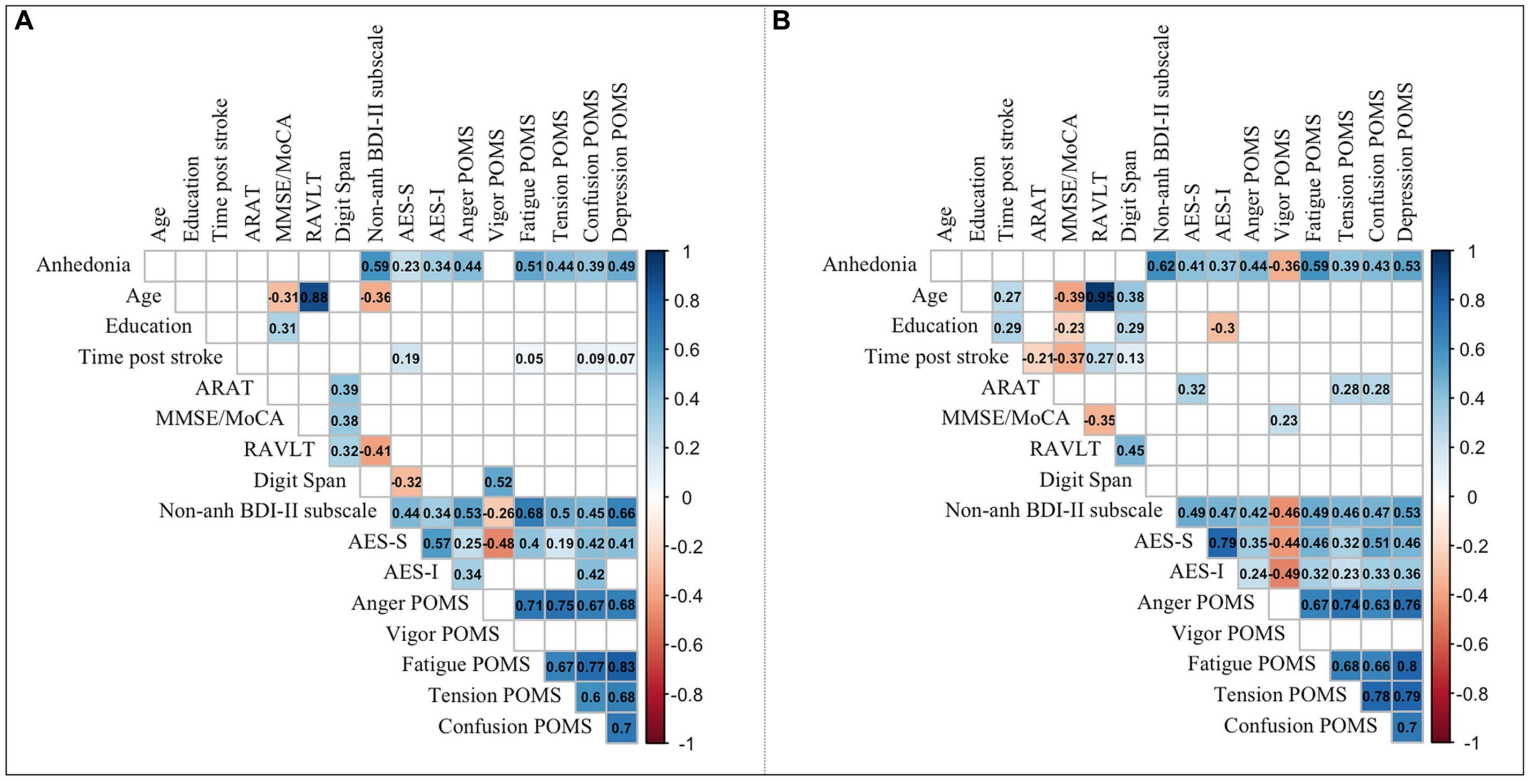
Correlations between demographic, clinical, and emotional variables. Significant correlation coefficients are shown for **(A)** subacute stroke patients, and **(B)** chronic stroke patients. *p* < 0.05. ARAT, Action Research Arm Test; MoCA, Montreal Cognitive Assessment; MMSE, Mini-Mental State Examination; RAVLT, Rey Auditory Verbal Learning Test; BDI-II, Beck Depression Inventory-II; AES-S, Self-Rated Version of the Apathy Evaluation Scale; AES-I, Informant Version of the Apathy Evaluation Scale; POMS, Profile of Mood States; POMS subscales: Anger-Hostility, Vigor-Activity, Fatigue-Inertia, Tension-Anxiety, Confusion-Bewilderment, Depression-Dejection.

Correlation analyses were applied to explore the association between anhedonia and the continuous demographic, clinical and emotional variables in SS and CS patients (see Figure 3; Supplementary Table S2). In both groups, anhedonia correlated positively with non-anhedonic depression BDI-II subscale and apathy scales (AES-S and AES-I), being AES-S more strongly correlated in CS than in SS patients (see Supplementary Figure S1). Moreover, anhedonia correlated with most POMS subscales, except for Vigor-Activity, which correlated negatively only in CS patients (see Figure 3; Supplementary Figure S2). The variable that correlated most strongly with the anhedonia in both groups was the non-anhedonic depression BDI-II subscale, followed by Fatigue-Inertia, Depression-Dejection, and Anger-Hostility of POMS. In CS patients, anhedonia correlated positively higher with AES-S compared to SS patients.

**Figure 4:**
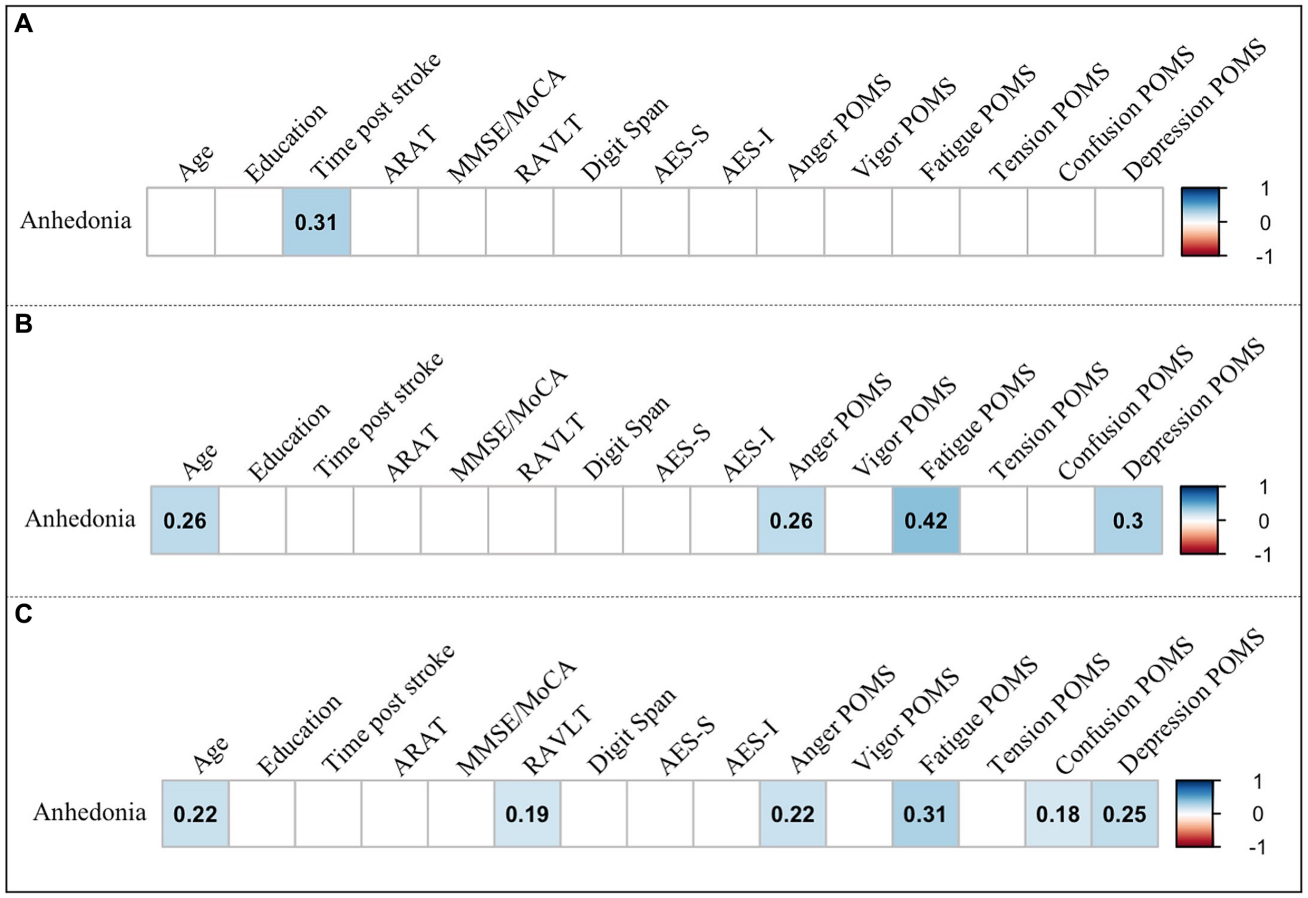
Partial correlations of anhedonia with demographic, clinical, and emotional variables. Significant correlation coefficients are shown for **(A)** subacute stroke, **(B)** chronic stroke, and **(C)** all stroke patients. *p* < 0.05. ARAT, Action Research Arm Test; MoCA, Montreal Cognitive Assessment; MMSE, Mini-Mental State Examination; RAVLT, Rey Auditory Verbal Learning Test; AES-S, Self-Rated Version of the Apathy Evaluation Scale; AES-I, Informant Version of the Apathy Evaluation Scale; POMS, Profile of Mood States; POMS subscales: Anger-Hostility, Vigor-Activity, Fatigue-Inertia, Tension-Anxiety, Confusion-Bewilderment, Depression-Dejection.

Partial correlation analyses were applied to explore the relationship between the anhedonia and the continuous demographic, clinical and emotional variables while controlling for the influence of the non-anhedonic depression BDI-II subscale in subacute and chronic stroke patients, and in the whole sample of patients (see Figure 4; Supplementary Table S3). In SS patients, the anhedonia score correlated positively with time post-stroke (measured in days). By contrast, in CS patients, anhedonia positively correlated with patients’ age and three subscales of POMS: Anger-Hostility, Fatigue-Inertia, and Depression-Dejection. When looking at the whole sample of stroke patients, anhedonia correlated positively with age and four subscales of POMS: Anger-Hostility, Fatigue-Inertia, Confusion-Bewilderment, and Depression-Dejection.

After comparing anhedonia level between stroke patients and HC while controlling for the confounding effects of the other emotional outcomes, we observed significant differences between stroke and healthy samples (*t*(189.72) = 5.95; *p* < 0.001; anhedonia mean score in stroke patients = 2.9 ± 2.3; anhedonia mean score in healthy controls = 1.3 ± 1.4).

## 4 Discussion

Anhedonia is an enduring behavioral trait characterized by a lack of reactivity to pleasurable stimuli (Der-Avakian and Markou, 2012). Although anhedonia has been associated with high levels of depression at hospital discharge post-stroke, no previous studies have explored its prevalence in subacute and chronic stroke patients with both ischemic and hemorrhagic etiologies (Sibon et al., 2012). We conducted an exploratory cohort study to investigate the prevalence and level of anhedonia in stroke survivors with upper-limb motor deficits in the subacute and chronic phases of the disease covering a wide range of time since stroke, from 1 month to 17 years post-stroke. We examined demographic, clinical, cognitive, motor, and emotional factors that could be related to post-stroke anhedonia. The exploratory nature of the study aimed to check the usefulness and importance of assessing anhedonia and related factors in the stroke population at both early and late stages of the disease (Hallingberg et al., 2018).

Our results clearly showed that people who had suffered a stroke were significantly more anhedonic than people who never suffered a stroke with similar age and years of education. In both subacute and chronic stroke patients, nearly 18–20% of individuals showed a higher level of anhedonia, whereas only 4.3% of healthy controls. Importantly, studies on chronic pain and other inflammatory conditions have observed similar results (Lucido et al., 2021). For example, Garland et al. (2020) reported that nearly 25% of chronic pain patients exhibited higher anhedonia levels, which is in line with previous research demonstrating higher anhedonia levels in women suffering endometric chronic pelvic pain (Mallorqui et al., 2022). Additionally, a recent review revealed the relationship of acute and chronic stress with increased peripheral and central inflammation and consequent dysregulation of the reward system, leading to the onset or development of anhedonia (Boyle et al., 2023).

Patients with both subacute and chronic stroke showed similar levels of anhedonia regardless of stroke etiology, time since stroke, affected hemisphere, lesion location, and levels of motor and cognitive functions. In contrast to previous studies that found a correlation between stroke in the left frontal cortex or left basal ganglia and the risk for PSD (Starkstein and Robinson, 1989), the present results are in line with other studies that did not support the influence of the affected hemisphere and the exact lesion location on the development of emotional and mood disorders (Carson et al., 2000; Colita et al., 2023). Our findings align with a neurophysiological explanation based on the inflammatory effect on brain functioning, which has been increasingly recognized as a key factor in the onset or development of several psychiatric disorders, including depression (Hassamal, 2023). When an ischemic or hemorrhagic stroke occurs, it results in neuronal cell death and the release of cytokines that elicit localized inflammation in the damaged brain region, among other neurochemical events (Brouns and De Deyn, 2009; Fann et al., 2013). At 6 months post-stroke, in the chronic phase of the disease, inflammatory cytokines levels have been found to decrease in the infarct zone and increase in more distal ipsilateral and contralateral brain areas (Pascotini et al., 2015; Hou et al., 2021; Stuckey et al., 2021). Notably, neuroinflammatory cytokines provoke a decrease in dopamine and an increase in glutamate concentrations, reducing the functional connectivity between the ventral striatum and prefrontal cortex (Felger et al., 2016). The altered neurotransmission in the reward system would result in abnormal reward valuation, abnormal calculation of required effort, and deficits in decision-making for optimal reward-based actions, thus affecting hedonic capacity (Der-Avakian and Markou, 2012; Treadway et al., 2012; Cooper et al., 2018).

Although the state of neuroinflammation after several years post-stroke remains unknown, previous studies on ischemic stroke demonstrated that inflammatory cytokines released in the acute phase can lead to tissue damage and continued cell death in the injury site and penumbra (Tobin et al., 2014; Pascotini et al., 2015). This process has been associated with a more severe, prolonged, and treatment-resistant course of mood disorders (Hassamal, 2023). Hence, the chronic low-grade endogenous inflammation could continuously shape the pathology following stroke, promoting the ongoing impairment of the mesolimbic pathway in the long term regardless of lesion location, stroke etiology, and time since stroke (Shi et al., 2019; Stuckey et al., 2021). Additionally, these impairments in top-down cognitive-behavioral processes could alienate patients from natural and previously acquired resources of pleasure and positive experiences, potentially contributing to anhedonia development even years after stroke (Mallorqui et al., 2022).

Individuals with anhedonia are characterized by enjoying less of things in life by themselves and with others, which triggers a lack of interest in engaging in rewarding activities and participating in community life in the long term (Kusec et al., 2019). In the stroke population, this could affect the desire and motivation to initiate and complete interventions at various steps of the neurorehabilitation process, thus decreasing the likelihood of potential recovery. Engaging in rehabilitation programs has been strongly associated with improving motor, neurocognitive, psychological, biological, and socio-environmental outcomes (Maclean and Pound, 2000; Siegert and Taylor, 2004). In this vein, a negative loop could be installed in which high levels of anhedonia impede recovery, leading to a decrease in patients’ autonomy and emotional well-being, which in turn could increase anhedonia levels. In our results, stroke patients demonstrated significantly lower motor and cognitive functions than healthy controls, while no differences were found between subacute and chronic groups. Based on prior evidence suggesting a correlation between moderate or severe depression and poor functional outcomes in both subacute and chronic stroke phases (Pohjasvaara et al., 2001), we expected to find a correlation between anhedonia and functional impairments. However, no associations were observed between any functional outcome and the level of anhedonia and non-anhedonic depression in both groups of patients (see Supplementary Tables S1–S3). While improved mood has been correlated with a greater cognitive enhancement in depressed patients (Murata et al., 2000), the influence of PSD on motor recovery is not clear. Some studies found no difference in motor improvement between depressed and non-depressed patients (Nannetti et al., 2005), while others suggested a poor motor recovery in non-treated depressed stroke patients (Gainotti et al., 1997). In this vein, larger-scale longitudinal studies testing the score change in functional outcomes would be needed to explore the influence of post-stroke anhedonia on functional outcomes recovery.

Most stroke survivors must cope with a sudden reduction of their autonomy and QoL, a context that can trigger the onset or development of mood disorders such as depression, often manifesting with symptoms such as anhedonia (Der-Avakian and Markou, 2012; Feigin et al., 2017; Marcheschi et al., 2018). As expected, anhedonia post-stroke was associated with a lack of motivation and negative mood states. Both groups of patients were significantly more apathetic than healthy controls. Moreover, apathy was more strongly correlated with anhedonia in chronic patients. Importantly, the decrease in affection, enthusiasm, and interest caused by apathy has been associated with delayed rehabilitation, reduced social interaction, and increased caregiver burden, affecting the QoL of patients and their relatives (Åström et al., 1992; Hama et al., 2007). Anhedonia was also strongly related to negative mood states such as fatigue-inertia, and to a lesser extent, anger-hostility, depression-dejection, tension-anxiety, and confusion-bewilderment in both groups of patients. Only the chronic group showed a decreased sense of vigor-activity related to anhedonia, suggesting the long-term negative impact of anhedonia on engaging in a healthy active lifestyle. These results are in line with a recent previous study suggesting the role of anhedonia as a predictor of somatic symptoms of depression, such as fatigue, over the first year after discharge from rehabilitation (Ashaie et al., 2023). Our findings also underlines the likely role of anhedonia in generating a deteriorating looping effect at a behavioral level (Mallorqui et al., 2022). Additionally, older patients in the chronic group exhibited higher levels of anhedonia, consistent with previous research suggesting that the ageing brain is more sensitive to neurodegeneration mechanisms (Yan et al., 2010; Li et al., 2020; Stuckey et al., 2021). Consequently, older patients with stroke would be at a higher risk of suffering from anhedonia in the long term, not only due to the greater likelihood of social isolation (Yeh and Lo, 2004), but also because of the detrimental neurophysiological effects provoked by the brain injury. Crucially, anhedonia was significantly higher in stroke patients compared to healthy controls when controlling for the confounding effects of depression, apathy, fatigue, anger, vigor, tension, and confusion despite its strong correlation with them. This suggests the individual role of anhedonia as an emotional condition that can appear or increase in individuals after suffering a stroke.

The present study has some limitations. First, the results cannot be extrapolated to all stroke patients, as the sample was selected for its recovery potential to participate in intensive post-stroke rehabilitation programs. Therefore, the data we explored belong to a group of patients with a higher motivation and activity level than the entire stroke population. Moreover, due to the exploratory nature of this study, our sample of stroke patients was small and limited to those with upper-limb motor deficits. Considering that stroke patients also experience other neurological and cognitive impairments such as aphasia, execution dysfunction or memory disorders (Grönberg et al., 2022; Cramer et al., 2023), our inclusion criteria made the results not representative of the entire stroke population. However, motor impairment remains one of the most prevalent consequences after stroke, with approximately half of survivors experiencing impaired upper-limb movements in the chronic phase of the disease (Lee et al., 2015; Ingram et al., 2021). Additionally, the clinical characterization of lesion location was overly broad, constraining the extent to which meaningful conclusions can be drawn regarding the impact of this variable on the development of anhedonia. Lastly, due to the difficulty of recruiting patients with identical demographic and clinical characteristics, the stroke groups were unbalanced in terms of sex and lesion location, which could affect the association of these factors with anhedonia.

## 5 Conclusion

This exploratory cohort study provides valuable insights about the prevalence, level, and factors related to anhedonia in the stroke population. The results reveal an important increase in anhedonia among individuals with ischemic and hemorrhagic stroke and at both subacute and chronic stages compared to a sample of healthy individuals. Crucially, patients exhibited similar levels of anhedonia regardless of stroke etiology, time since stroke, affected hemisphere, lesion location, and levels of motor and cognitive functions. Furthermore, anhedonia was associated with a lack of motivation and higher levels of negative mood states such as fatigue and anger, thereby reducing emotional well-being and QoL, and presumably leading to a potential decrease in engagement with rehabilitation programs. Importantly, anhedonia levels were significantly superior in stroke patients compared to healthy controls when controlling for the confounding effects of depression, non-anhedonic depression, apathy, fatigue, anger, vigor, confusion, and tension, suggesting its individual role in the stroke population. Anhedonia has been identified as a stable trait over time with poor remission despite pharmacological, psychological or neurostimulation treatments (Shankman et al., 2010; Pizzagalli, 2022). For this reason, more research is needed to explore the presence of anhedonia in large-scale cohort and longitudinal studies, including stroke patients with other functional consequences, to test its influence on long-term functional and emotional recovery. Lastly, studying the prevalence of anhedonia in patients with other neurological and inflammatory diseases would contribute to a better understanding of the etiology of this condition and help develop more effective behavioral interventions to incorporate its treatment into stroke rehabilitation programs.

## Data availability statement

The raw data supporting the conclusions of this article will be made available by the authors, without undue reservation.

## Ethics statement

The studies involving humans were approved by ethics committees of Bellvitge University Hospital and Hospital del Mar. The studies were conducted in accordance with the local legislation and institutional requirements. Written informed consent for participation in this study was provided by the participants’ legal guardians/next of kin.

## Author contributions

AR-F and JG-S conceived the study. AR-F, JG-S, and ES designed the study. ED, JG-S, and ES recruited the participants. ES, JG-S, and MP conducted and corrected the evaluations. ES and AV-B analyzed the data. ES, AM, and AR-F wrote the manuscript. All authors contributed to the article and approved the submitted version.
