# Supplementary material for: The presence of anhedonia in individuals with subacute and chronic stroke: an exploratory cohort study
Source: Front Aging Neurosci. 2024 Feb 7;16:1253028. doi: 10.3389/fnagi.2024.1253028 (PMC10880106; doi:10.3389/fnagi.2024.1253028)
Supplement: Supplementary file 1 [file Data_Sheet_1.pdf]

Supplementary material

1 Supplementary Figures and Tables

1.1 Supplementary Figures

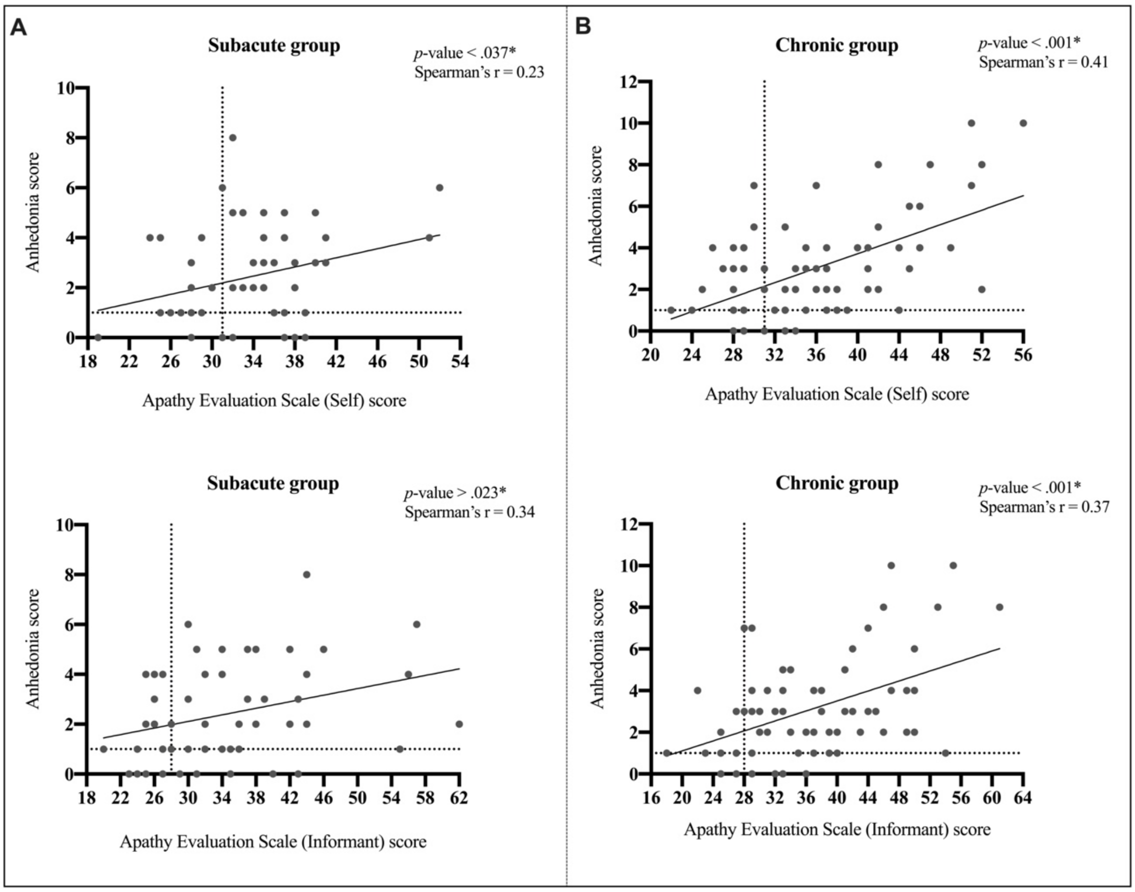

Supplementary Figure 1. Correlations plots of anhedonia with apathy scales. These graphics show the correlation between the anhedonia score and self- and informant-versions of apathy scores for A) subacute stroke, and B) chronic stroke.

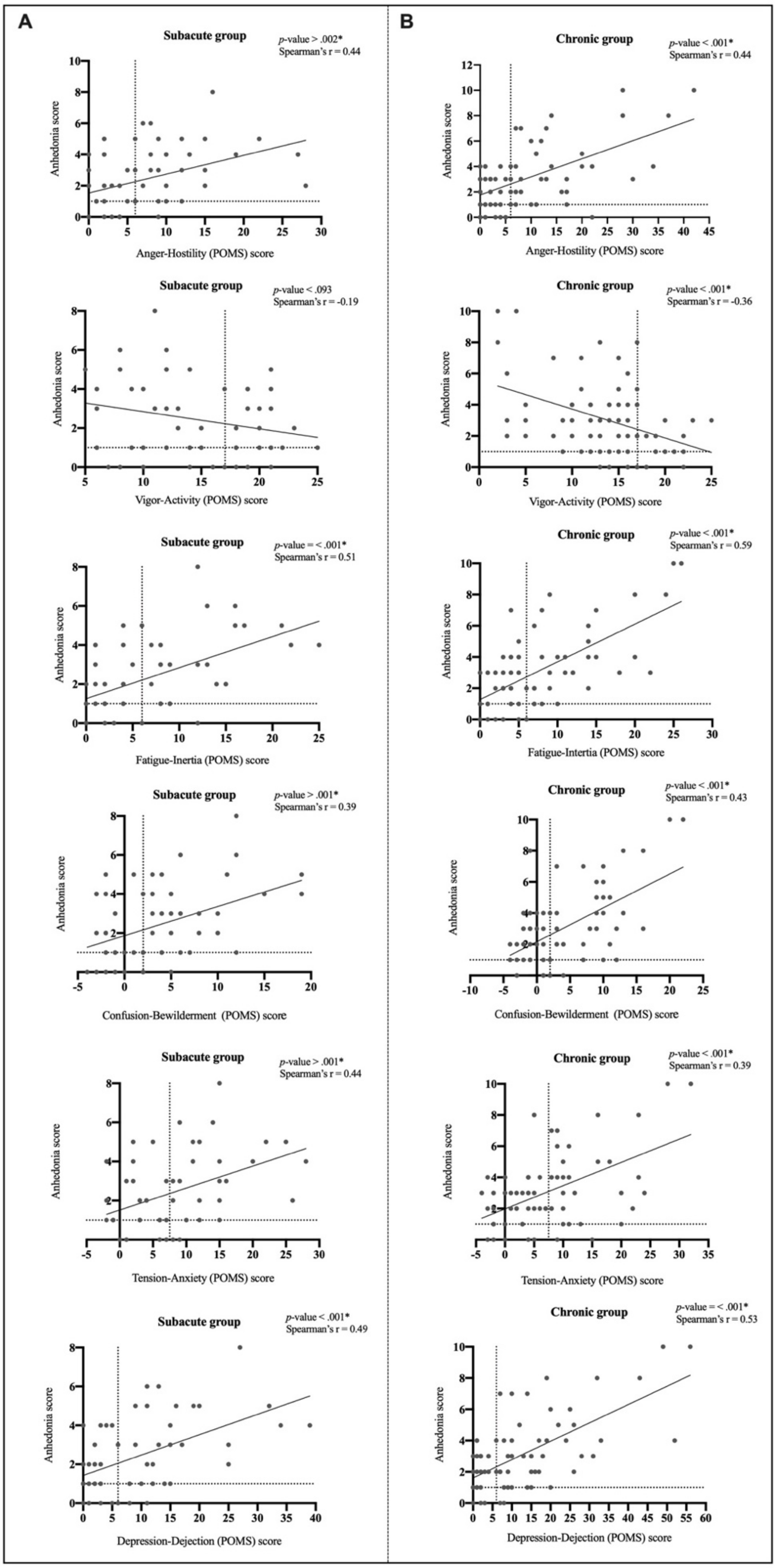

Supplementary Figure 2. Correlations plots of anhedonia with POMS. These graphics show the correlation between anhedonia scores and all POMS subscales scores for A) subacute stroke, and B) chronic stroke. (POMS: Profile of Mood States).

1.2 Supplementary Tables

Supplementary Table 1. **Descriptive analysis for the groups of patients classified into higher and lower levels of anhedonia.** Absolute frequencies and percentages are shown for sex, rehabilitation phase, stroke etiology, affected hemisphere, lesion location, cognitive level, depression level, non-anhedonic depression level, and presence of apathy. The mean and the standard deviation (SD) or the median and interquartile ranges [IQR] are shown for the quantitative variables with parametric and nonparametric distribution respectively. $p < 0.05$. (HAnh: Higher Anhedonia; LAnh: Lower Anhedonia; HDep: Higher non-anhedonic Depression; LDep: Lower non-anhedonic Depression; ARAT: Action Research Arm Test; MoCA: Montreal Cognitive Assessment; MMSE: Mini-Mental State Examination; RAVLT: Rey Auditory Verbal Learning Test; BDI-II: Beck Depression Inventory-II; AES-S: Self-Rated Version of the Apathy Evaluation Scale; AES-I: Informant Version of the Apathy Evaluation Scale; POMS: Profile of Mood States; Mild impairment < 27 in MMSE and < 26 in MoCA; None depression: 0-9 scores in BDI-II; Mild depression: 10-18 scores in BDI-II; Moderate depression: 19-29 scores in BDI-II; Severe depression: 30-63 scores in BDI-II; Presence of apathy ≥ 34 in AES-S and AES-I; Higher anhedonia > 4 in anhedonia BDI-II subscale; Lower anhedonia ≤ 4 in anhedonia BDI-II subscale; Higher non-anhedonic depression > 16.65 in non-anhedonic depression BDI-II subscale; Lower non-anhedonic depression ≤ 16.65 in non-anhedonic depression BDI-II subscale).

	**HAnh**<br>**N = 24**	**LAnh**<br>**N = 101**	***p*-value**
**Demographic variables**			
Age	60.2 (11.05)	62.3 (10.13)	.369
Sex			
Females	10 (41.67 %)	31 (30.70 %)	.338
Males	14 (58.33 %)	70 (69.30 %)	
Education level			
Years of education	15.5 [4.75]	18 [7]	.023*
**Clinical variables**			
Rehabilitation phase			
Subacute	10 (41.67 %)	44 (43.56 %)	>. 999
Chronic	14 (58.33 %)	57 (56.43 %)	
Stroke etiology			
Ischemic	15 (62.50 %)	73 (72.28 %)	.317
Hemorrhagic	9 (37.50 %)	28 (27.72 %)	
Affected hemisphere			
Right	11 (45.83 %)	50 (49.50 %)	.822
Left	13 (54.17 %)	51 (50.50 %)	
Lesion location			
Cortical	2 (8.33 %)	10 (9.90 %)	>. 999
Cortico-subcortical	11 (45.83 %)	39 (38.61 %)	
Subcortical	9 (37.50 %)	38 (37.62 %)	
Brainstem	1 (4.17 %)	8 (7.92 %)	
Cerebellum	1 (4.17 %)	6 (5.94 %)	
Time since stroke			
Months post stroke	7.5 [18.75]	9.5 [23.50]	.745
Motor ability			

ARAT	42 [20.50]	42 [17]	.940
Cognitive level			
MoCA/MMSE	27 [5]	27 [4]	.762
No impairment (%)	15 (62.50 %)	62 (61.39 %)	>. 999
Mild impairment (%)	9 (37.50 %)	39 (38.61 %)	
Memory			
Digit span (normative)	10.17 (3.27)	11.22 (2.91)	.123
RAVLT (normative)	34.25 (9.06)	37.67 (11.35)	.172
**Emotional variables**			
Anhedonia			
Anhedonia score	6 [3]	2 [2]	< .001*
Depression			
BDI-II score	23.5 [13.25]	8 [8]	< .001*
None	0 (0 %)	58 (57.43 %)	< .001*
Mild	7 (29.17 %)	36 (35.64 %)	
Moderate	9 (37.50 %)	7 (6.93 %)	
Severe	8 (33.33 %)	0 (0 %)	
Non-anhedonic depression			
Non-anhedonic BDI-II score	7 [9]	8 [8]	.676
Lower non-anhedonic depression	19 (79.17 %)	86 (85.14 %)	.536
Higher non-anhedonic depression	5 (20.83 %)	15 (14.85 %)	
Apathy informed by patient			
AES-S score	40 [16]	34 [9]	< .001*
Presence	16 (69.57 %)	56 (55.45 %)	.249
No presence	7 (30.43 %)	45 (44.55 %)	
Apathy informed by caregiver			
AES-I score	43 [13.75]	33 [11]	< .001*
Presence	19 (79.17 %)	50 (49.50 %)	.011*
No presence	5 (20.83 %)	51 (50.50 %)	
POMS			
Anger-Hostility score	12 [9.5]	5 [7]	< .001*
Vigor-Activity score	10.52 (5.28)	14.71 (5.01)	< .001*
Fatigue-Inertia score	14 [10]	4 [7]	< .001*
Tension-Anxiety score	12 [8.5]	5 [9]	< .001*
Confusion-Bewilderment score	10 [7]	2 [7]	< .001*
Depression-Dejection score	20 [14]	6 [11]	< .001*

Note: All participants completed the whole evaluation, except for one participant in the subacute group who did not respond the AES-S and POMS and did not provide SS the AES-I. Spouses mainly answered the AES-I, followed by siblings, children, and caregiver.

Supplementary Table 2. Correlation analysis of anhedonia with demographic, clinical, and emotional variables. This table shows the Spearman's *rho* and *p*-value of the correlations between the anhedonia score and the demographic, clinical, and emotional continuous variables score. $p < 0.05$. (ARAT: Action Research Arm Test; MMSE: Mini-Mental State Examination; MoCA: Montreal Cognitive Assessment; RAVLT: Rey Auditory Verbal Learning Test; AES-S: Self-Rated Version of the Apathy Evaluation Scale; AES-I: Informant Version of the Apathy Evaluation Scale; BDI-II: Beck Depression Inventory-II; POMS: Profile of Mood States).

	**Subacute stroke**		**Chronic stroke**	
	**Spearman's *rho***	***p*-value**	**Spearman's *rho***	***p*-value**
**Demographic variables**				
Age	-0.08	< .552	0.09	< .439
Education	-0.26	< .057	-0.06	> .609
**Clinical variables**				
Time post stroke	0.27	> .051	0.11	> .358
Motor function				
ARAT	0.02	> .869	-0.02	> .868
Cognitive functions				
MMSE/MoCA	-0.02	< .885	-0.19	< .122
RAVLT	-0.10	< .491	0.10	< .419
Digit Span	-0.13	> .367	0.06	> .600
**Emotional variables**				
Non-anhedonic BDI-II subscale	0.59	< .001*	0.62	< .001*
AES-S	0.23	< .037*	0.41	< .001*
AES-I	0.34	> .023*	0.37	< .001*
Anger-Hostility (POMS)	0.44	> .002*	0.44	< .001*
Vigor-Activity (POMS)	-0.23	< .093	-0.36	< .001*
Fatigue-Intertia (POMS)	0.51	< .001*	0.59	< .001*
Tension-Anxiety (POMS)	0.44	> .001*	0.39	< .001*
Confusion-Bewilderment (POMS)	0.39	> .001*	0.43	< .001*
Depression-Dejection (POMS)	0.49	< .001*	0.53	< .001*

Supplementary Table 3. **Partial correlation analysis of anhedonia with demographic, clinical, and emotional variables.** Spearman's *rho* and *p*-value correlations between anhedonia score and the continuous demographic, clinical, and emotional variables score are shown for both groups of stroke patients and all patients together. $p < 0.05$. (ARAT: Action Research Arm Test; MMSE: Mini-Mental State Examination; MoCA: Montreal Cognitive Assessment; RAVLT: Rey Auditory Verbal Learning Test; AES-S: Self-Rated Version of the Apathy Evaluation Scale; AES-I: Informant Version of the Apathy Evaluation Scale; BDI-II: Beck Depression Inventory-II; POMS: Profile of Mood States).

	**Subacute stroke**		**Chronic stroke**		**All patients**	
	**Spearman's *rho***	***p*-value**	**Spearman's *rho***	***p*-value**	**Spearman's *rho***	***p*-value**
**Demographic variables**						
Age	0.21	< .126	0.26	< .031*	0.22	< .014*
Education	-0.20	< .161	0.06	< .643	-0.05	> .565
**Clinical variables**						
Time post stroke	0.31	< .025*	0.07	< .560	0.16	< .079
Motor function						
ARAT	0.11	< .451	-0.09	> .450	0.02	< .832
Cognitive functions						
MMSE/MoCA	0.19	< .188	-0.21	> .078	-0.09	> .327
RAVLT	0.20	> .152	0.21	> .080	0.19	< .033*
Digit Span	0.04	< .765	0.08	< .502	0.07	< .436
**Emotional variables**						
AES-S	-0.04	< .775	0.15	< .210	0.08	> .354
AES-I	0.18	< .210	0.13	> .302	0.15	< .111
Anger-Hostility (POMS)	0.18	< .211	0.26	< .033*	0.22	> .015*
Vigor-Activity (POMS)	-0.05	< .706	-0.11	> .372	-0.10	< .273
Fatigue-Intertia (POMS)	0.19	< .182	0.42	< .001*	0.31	< .001*
Tension-Anxiety (POMS)	0.20	> .153	0.16	> .190	0.15	< .091
Confusion-Bewilderment (POMS)	0.17	< .240	0.21	> .085	0.18	< .048*
Depression-Dejection (POMS)	0.17	< .240	0.30	> .012*	0.25	< .006*
